# Is spontaneous echo contrast associated with device-related thrombus or embolic events after left atrial appendage occlusion? - Insights from the multicenter German LAARGE registry

**DOI:** 10.1007/s10840-023-01567-z

**Published:** 2023-06-01

**Authors:** Christian Fastner, Claus Müller, Johannes Brachmann, Thorsten Lewalter, Ibrahim Akin, Horst Sievert, Matthias Käunicke, Uwe Zeymer, Matthias Hochadel, Steffen Schneider, Jochen Senges, Damir Erkapic, Christian Weiß

**Affiliations:** 1 Department of Cardiology, Angiology, Haemostaseology and Medical Intensive Care, University Medical Centre Mannheim, Medical Faculty Mannheim, Heidelberg University, European Center for AngioScience (ECAS), and German Center for Cardiovascular Research (DZHK) Partner Site Heidelberg/Mannheim, Theodor-Kutzer-Ufer 1-3, 68167 Mannheim, Germany; 2 Department of Cardiology, Städtisches Klinikum Lüneburg gGmbH, Lüneburg, Germany; 3 REGIOMED‐Kliniken, Coburg, Germany and University of Split, School of Medicine, Split, Croatia; 4 Department of Medicine, Cardiology and Intensive Care, Hospital Munich‐Thalkirchen, Munich, Germany; 5 CardioVascular Center (CVC) Frankfurt, Frankfurt, Germany; 6 Department of Cardiology, University of Witten/Herdecke, Katholisches Klinikum Essen, Essen, Germany; 7 Department of Cardiology, Klinikum Ludwigshafen, Ludwigshafen, Germany; 8 Stiftung Institut Für Herzinfarktforschung, Ludwigshafen, Germany; 9 Department of Cardiology, Rhythmology and Angiology, Diakonie Klinikum Siegen, Siegen, Germany and Department of Cardiology and Angiology, University Hospital Giessen, Giessen, Germany

**Keywords:** Atrial fibrillation, Thromboembolism, Ischemic stroke, Bleeding risk, Interventional approach, Left atrial appendage closure

## Abstract

**Background:**

Interventional left atrial appendage occlusion (LAAO) provides an alternative to oral anticoagulation (OAC) for prophylaxis of thromboembolic events (TEs) in nonvalvular atrial fibrillation patients, predominantly in those with high bleeding risk and contraindications for long-term OAC. Although spontaneous echo contrast (SEC) is a well-known risk factor for atrial thrombus formation, little is known about whether this means an increased risk of device-related thrombus (DRT) or TEs following LAAO.

**Methods:**

This substudy of the prospective, multicenter German LAARGE registry assessed two groups according to absence (SEC −) or presence of SEC (SEC +) in preprocedural cardiac imaging. Clinical and echocardiographic parameters were registered up to 1 year after LAAO.

**Results:**

Five hundred eighty-eight patients (SEC − 85.5 vs. SEC + 14.5%) were included. More SEC + patients were implanted for OAC non-compliance (11.8 vs. 4.6%, *p* = 0.008) and a higher proportion received only antiplatelet therapy without OAC at hospital discharge (96.5 vs. 86.0%, *p* = 0.007). The SEC + patients had larger LA diameters (50 (47; 54) vs. 47 (43; 51) mm, *p* < 0.001), wider LAA ostia (21 (19; 23) vs. 20 (17; 22) mm at 45°, *p* = 0.011), and lower left ventricular ejection fraction (50 (45; 60) vs. 60 (50; 60) %, *p* < 0.001) on admission. Procedural success was very high in both groups (98.1%, *p* = 1.00). Periprocedural major adverse cardiac and cerebrovascular events and other major complications were rare in both groups (3.8 vs. 4.7%, *p* = 0.76). At follow-up, DRT was only detected in the SEC − group (3.8 vs. 0%, *p* = 1.00). The rates of TEs (SEC − 1.2 vs. SEC + 0%, *p* = 1.00) after hospital discharge and 1-year mortality (SEC − 12.0 vs. SEC + 11.8%, *p* = 0.96) were not significantly different between the two groups.

**Conclusions:**

Presence of SEC at baseline was not associated with an increased rate of DRT or TEs at 1-year follow-up after LAAO in LAARGE.

**Supplementary Information:**

The online version contains supplementary material available at 10.1007/s10840-023-01567-z.

## Introduction

Thromboembolic events (TEs) are causal for nonvalvular atrial fibrillation (AF)–related morbidity and mortality [1]. In particular, cerebrovascular embolism is associated with poor outcome and immense costs to health care systems [2]. Oral anticoagulation (OAC) is the therapy of choice to prevent these serious AF complications, preferably with non-vitamin K OAC (NOAC) [3]. However, in a substantial number of patients, there are contraindications for long-term OAC [4]. Left atrial appendage occlusion (LAAO) is an appropriate alternative for these patients and is nowadays routinely used worldwide [5]. The devices follow a plug or pacifier principle, with either a convex lobe or a disc sealing the LAA from the left atrium [6]. After implantation, thrombus formation on the occluder’s surface (i.e., device-related thrombosis, DRT) is a rare complication, but it is associated with a four- to fivefold increase in ischemic events [7]. DRT is predominantly detected in the first year after implantation, and the incidence is thought to be as high as 10% [6, 7]. The expert consensus statement of the European Heart Rhythm Association recommends cardiac imaging within six months after implantation, which may be repeated after twelve months, to detect DRT [6]. Several predictors for increased risk of DRT are reported: patient-related, these include larger LAA diameter, reduced left ventricular ejection fraction (LVEF), permanent AF, higher CHA_2_DS_2_-VASc score, prior TE, or early discontinuation/incompliance with antithrombotic medication, and implantation-related, deep occluder implantation or incomplete occlusion of the LAA [8–10].

Spontaneous echo contrast (SEC) is an echocardiographic phenomenon in AF patients due to red blood cell aggregation in the LA(A) and is associated with increased TE rates [11, 12]. There is a large overlap in the risk factors leading to SEC and DRT [11]. It is therefore reasonable to assume that there might be an increased rate of DRT and consecutive TEs in those patients in whom SEC is detectable on preprocedural imaging before LAAO. We examined the study population of the Left-Atrium-Appendage occluder Register - GErmany (LAARGE; ClinicalTrials.gov Identifier: NCT02230748) with respect to the occurrence of DRT and/or TEs stratified by the detection of SEC at baseline.

## Methods

### Study design

Patients with nonvalvular AF receiving LAAO from 38 centers in Germany were included into the prospective, multicenter LAARGE between July 2014 and January 2016. Methods and main results were published previously [13]. The Institut für Herzinfarktforschung (IHF; Ludwigshafen am Rhein, Germany) was responsible for conducting the study. Participating centers were encouraged to include all LAAO patients consecutively to avoid a recruitment bias. Indication for LAAO was at the discretion of the center, and no specific inclusion or exclusion criteria were used in order to maintain the real-world character of the study population. Moreover, no default was given for the selection of the occluder type. For this substudy, all patients with started procedure, endovascular LAAO, and information on a preprocedural cardiac imaging were selected from the whole database. Documentation regarding the presence or absence of SEC in preprocedural cardiac imaging was explicitly required and submitted in an electronic case report form by the treating physician; grading of SEC density was not documented/performed [14]. The study population was divided into two groups according to atrial imaging findings at baseline: no SEC (i.e., SEC − group) versus (vs.) SEC present (i.e., SEC + group). Patients with LA(A) thrombus were excluded. In case of reported stroke, systemic embolism (SE), or major bleeding medical documents were requested from the respective site and centrally assessed and verified by an independent critical event committee. Prior to participation, informed consent was obtained from all patients. The study was approved by the ethics committee of the Landesärztekammer Rheinland‐Pfalz (Mainz, Germany) as well as local ethics committees of the participating centers and was performed in accordance with the Helsinki Declaration.

### Follow-up

Data up to hospital discharge were collected by the respective centers and reported to the IHF through an electronic case report form. The long-term follow-up was performed by the IHF using telephone interviews based on standardized questionnaires 12 months after LAAO. In addition to the central follow-up, the participating centers reported cases of death, complications and adverse events, clinical data, and echocardiographic exams. If patients could not be contacted, information was obtained from the registration offices.

### Definition of outcome measures

In this substudy, the primary outcome measure was the occurrence of DRT in comparison of groups. Secondarily, the effectiveness of LAAO was assessed by the combined absence of nonfatal stroke, transient ischemic attack (TIA), or SE in survivors after hospital discharge. In addition, the absence of all-cause death was considered. Rates of intra-hospital complications, device-related complications, or those potentially related to antithrombotic therapy in the first year were used to assess treatment safety.

Technical success was assumed in the presence of a stable device anchorage without paradevice leak > 5 mm. Severe bleeding was defined as hemodynamically unstable, requiring transfusion, requiring surgical treatment, or any intracranial hemorrhage. Moderate bleeding was defined as bleeding requiring medical intervention without meeting the above criteria for major bleeding.

### Statistical analysis

Continuous variables were expressed as median with interquartile range, and the risk scores as means with standard deviation. Categorical variables were expressed as absolute numbers and percentages. For the comparison of continuous variables between patient groups, Mann-Whitney-Wilcoxon test was used. Pearson’s chi-squared test was used for the comparison of categorical variables, and Fisher’s exact test in case of low event rates. One‐year mortality after hospital discharge was evaluated by means of survival analysis. These statistics are based on the available cases. Statistical significance was defined as *p* ≤ 0.05 (two‐tailed). Statistical analyses were performed with SAS® version 9.4 (SAS Institute, Cary, NC, USA).

## Results

### Study population

A total of 641 patients were enrolled in LAARGE. Of them, 588 (91.7%) were included in this LAARGE substudy: 503 (85.5%) in SEC − group, 85 (14.5%) in SEC + group. No statistically significant differences between both groups were seen with regard to age, sex, frequency of cardiovascular diseases, and risk scores (Table 1). However, the rate of nonischemic and nondilated cardiomyopathies was higher in the SEC + group (8.2 vs. 1.6%, *p* < 0.001). Of all patients, 60.2% received transthoracic, and 90.5% transesophageal echocardiography prior to the procedure, respectively (more than one imaging modality per patient in some cases, each *p* > 0.05 for comparison of groups). The SEC + patients had lower median LVEF of 50 (45; 60) vs. 60 (50; 60)% (*p* < 0.001), greater LA and LAA dimensions (each *p* < 0.005; supplementary table 1). There were marked differences in the indications for LAAO: The SEC + group had a higher rate of patients implanted due to OAC non-compliance (11.8 vs. 4.6%, *p* = 0.008), and the SEC − group had a higher rate of patients implanted due to adverse drug reactions (20.9 vs. 10.6%, *p* = 0.027). Most patients were screened for LAAO with prior bleeding (SEC − 81.3 vs. SEC + 72.9%, *p* = 0.074, of them SEC − 50.9 vs. SEC + 41.9% major bleedings). More SEC + patients presented with AC (74.1 vs. 60.0%, *p* = 0.013), and this was due to more cases with vitamin K antagonist (28.2 vs. 17.1%, *p* = 0.015). There was no statistically significant difference for antiplatelet agents (APA).

**Table 1:** Baseline characteristics of the study population

	SEC −	SEC +	*p* value*
Total cohort, *n* (% of all patients)	503 (85.5)	85 (14.5)	
Male sex, *n* (%)	308 (61.2)	53 (62.4)	0.84
Age [years], median (IQR)	77 (73; 81)	77 (73; 82)	0.73
Body mass index [kg/m^2^], median (IQR)	27 (24; 30)	26 (24; 31)	0.41
CHA_2_DS_2_-VASc score, mean ± SD	4.5 ± 1.5	4.8 ± 1.9	0.076
HAS-BLED score, mean ± SD	3.9 ± 1.1	3.8 ± 1.1	0.29
Arterial hypertension, *n* (%)	468 (93.0)	79 (92.9)	0.97
Diabetes mellitus, *n* (%)	167 (33.2)	36 (42.4)	0.10
Coronary heart disease, *n* (%)	233 (46.3)	44 (51.8)	0.35
Peripheral arterial disease, *n* (%)	139 (27.6)	20 (23.5)	0.43
eGFR (MDRD), median (IQR)	63 (42; 81)	57 (35; 74)	0.13
Type of atrial fibrillation, each *n* (%)			
• Paroxysmal	210 (41.7)	34 (40.0)	0.76
• Persistent	81 (16.1)	21 (24.7)	0.053
• Permanent	212 (42.1)	30 (35.3)	0.24
Atrial fibrillation upon hospital admission, *n* (%)	315 (62.6)	62 (72.9)	0.067
Prior pulmonary vein isolation, *n* (%)	12 (2.4)	5 (5.9)	0.075
Indication for LAAO, each *n* (%)			
• Prior bleeding	409 (81.3)	62 (72.9)	0.074
• Prior cerebrovascular event despite OAC	128 (25.4)	26 (30.6)	0.32
• Adverse drug reaction	105 (20.9)	9 (10.6)	**0.027**
• Labile INR	45 (8.9)	7 (8.2)	0.83
• Incompliance with OAC	23 (4.6)	10 (11.8)	**0.008**
• Patient’s preference	127 (25.2)	25 (29.4)	0.42
• Other reason	44 (8.7)	12 (14.1)	0.12

^*^Tested by Pearson’s *X*^2^ or Mann–Whitney-Wilcoxon test; bold indicates *p* < 0.05; more than one item could occur in the same patient; patient’s preference refers to the situation where the patient was recommended the use of conventional OAC yet rejected it for fear of severe bleeding; *eGFR* estimated glomerular filtration rate, *INR* international normalized ratio, *IQR* interquartile range, *LAAO* left atrial appendage occlusion, *OAC* oral anticoagulation, *SD* standard deviation

### Procedural details

Technical success was very high with SEC − 98.0 and SEC + 98.8% of patients after started procedure, respectively (*p* = not significant (NS); supplementary table 2). In only SEC − 0.4 vs. SEC + 1.2% of patients device-anchorage was not stable (*p* = NS). No paradevice leak > 5 mm was detected. In the SEC + group, more Amplatzer™ Cardiac Plugs (36.5 vs. 23.9%, *p* = 0.014) and Amplatzer™ Amulets™ (36.5 vs. 23.9%, *p* = 0.014; both Abbott, Chicago, IL, USA) and fewer WATCHMANs™ (22.4 vs. 50.6%, *p* < 0.001; Boston Scientific, Marlborough, MA, USA) were used. Total fluoroscopy time (SEC − 10 (7; 15) vs. SEC + 11 (8; 15) min) was not statistically significantly different between the groups.

### Intrahospital outcome

**Fig. 1:**
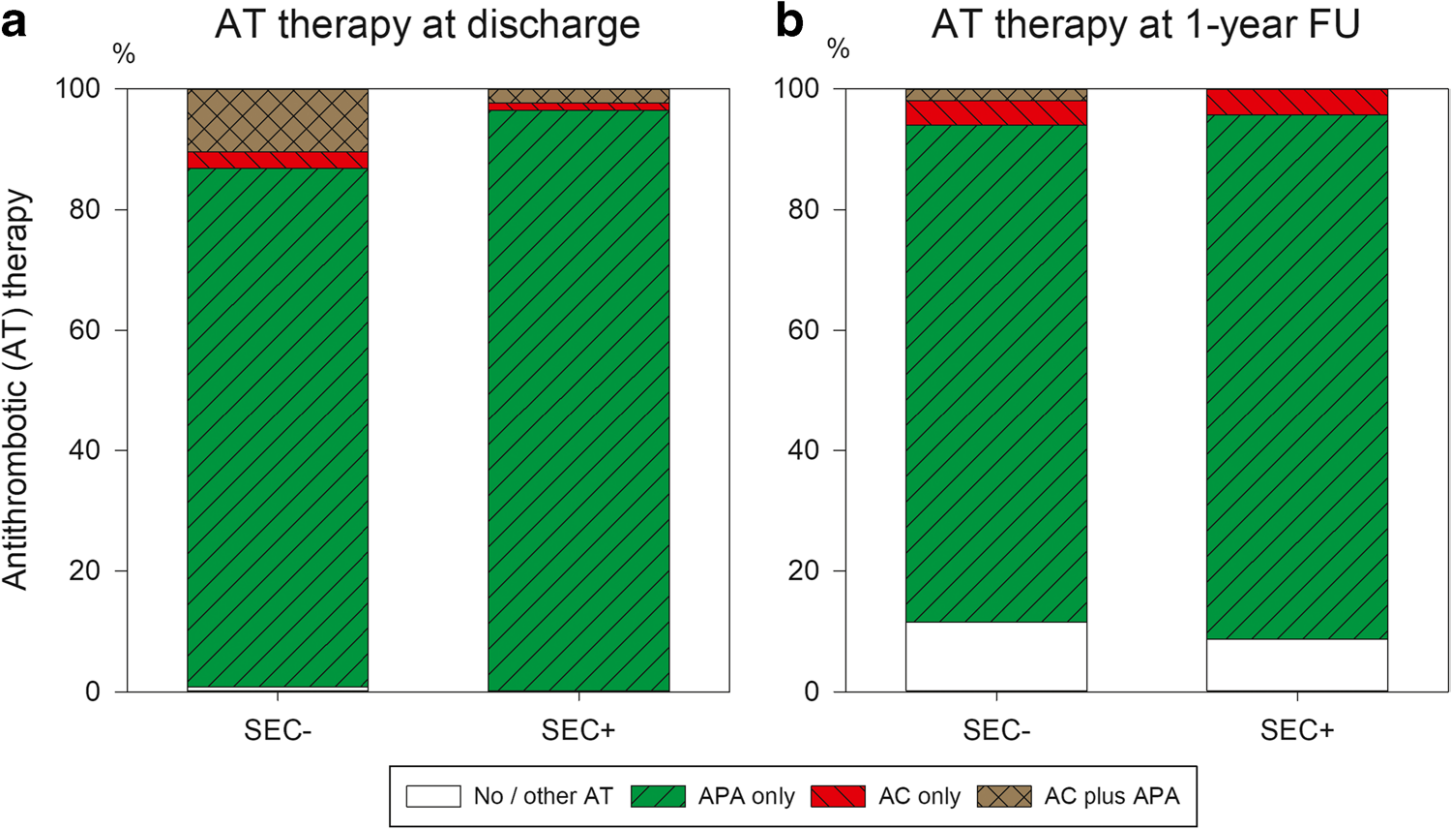
Antithrombotic medication at **a **hospital discharge and **b **1-year follow-up (FU) in patients without ( −) and with ( +) spontaneous echo contrast (SEC) at baseline; *AC *anticoagulation, *APA *antiplatelet agent

Intrahospital major adverse cardiac and cerebrovascular events (MACCE) were extremely rare in both groups (SEC − 0.6 vs. SEC + 0%, *p* = NS; Table 2). One case of death in the SEC − group was assessed as noncardiac and one in the same group as cardiac-related. During this period, only 1 stroke occurred in the SEC − group. Other severe and moderate complications were recorded as SEC − 3.2 vs. SEC + 4.7% (*p* = NS) and SEC − 9.1 vs. SEC + 9.4% (*p* = NS), respectively. Postprocedural length of stay did not differ statistically significantly between both groups, with a median of 2 days. At hospital discharge, more patients in the SEC − group received dual antithrombotic therapy of AC plus APA (10.4 vs. 2.4%, *p* = 0.0018; Fig. 1A), fewer only APA (86.0 vs. 96.5%, *p* = 0.007).

**Table 2:** Intrahospital outcome

	SEC −	SEC +	*p* value*
Total cohort, *n* (% of all patients)	503 (85.5)	85 (14.5)	
MACCE, *n* (%)	3 (0.6)	0 (0)	1.00
All-cause death, *n* (%)	2 (0.4)	0 (0)	1.00
Myocardial infarction, *n* (%)	1 (0.2)	0 (0)	1.00
Nonfatal stroke, *n* (%)	1 (0.2)	0 (0)	1.00
Other major complications, *n* (%)	16 (3.2)	4 (4.7)	0.51
Severe bleeding, *n* (%)	6 (1.2)	0 (0)	0.60
AV fistula or pseudoaneurysm, *n* (%)	3 (0.6)	1 (1.2)	0.47
Pericardial effusion requiring action, each *n* (%)			
• Surgical	1 (0.2)	0 (0)	1.00
• Interventional	7 (1.4)	3 (3.5)	0.16
Device dislodgement requiring action, each *n* (%)			
• Surgical	0 (0)	0 (0)	–-
• Interventional	2 (0.4)	0 (0)	1.00
Moderate complications, *n* (%)	46 (9.1)	8 (9.4)	1.00
Moderate bleeding, *n* (%)	8 (1.6)	1 (1.2)	1.00
Extended groin hematoma, *n* (%)	15 (3.0)	3 (3.5)	0.73
Access site infection, *n* (%)	1 (0.2)	0 (0)	1.00
Pericardial effusion with conservative treatment, *n* (%)	6 (1.2)	4 (4.7)	**0.043**
Device dislodgement handled by immediate retraction, *n* (%)	5 (1.0)	0 (0)	1.00
Transient ischemic attack, *n* (%)	0 (0)	0 (0)	–-
Successful cardiopulmonary resuscitation, *n* (%)	2 (0.4)	0 (0)	1.00

^*^Tested by Fisher’s exact test; bold indicates *p* < 0.05; more than one item could occur in the same patient; *AV* arteriovenous, *IQR* interquartile range, *MACCE* major adverse cardiac and cerebrovascular events

### Follow-up

**Fig. 2:**
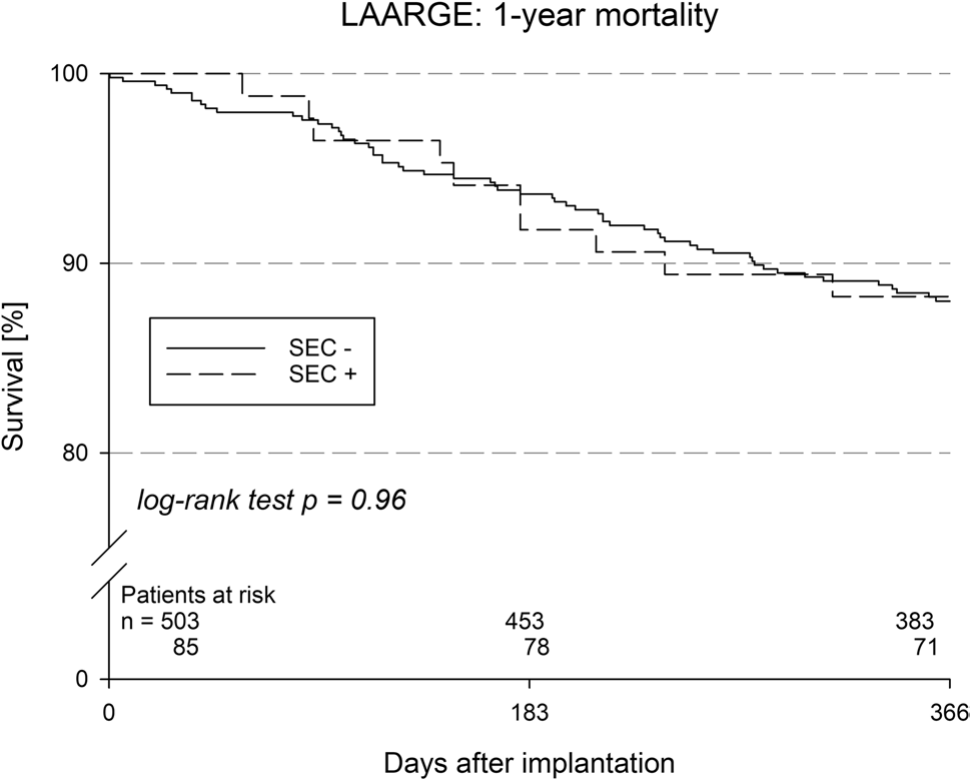
Kaplan–Meier analysis of 1-year all-cause mortality in patients without ( −) and with ( +) spontaneous echo contrast (SEC)

Follow-up imaging was performed at a median of SEC − 98 (50; 185) and SEC + 109 (31; 349) days after index discharge (*p* = NS) in SEC − 37.5 and SEC + 22.4% of patients, respectively. Information on vital status was obtained from SEC − 97.8% and all SEC + patients (Table 3). In SEC − 3.8 vs. SEC + 5.3% of patients, LA thrombi were detected (*p* = NS), of them in the SEC − group all 7 were connected to the occluder, i.e., DRT, and in the SEC + group 1 in the LA itself was not directly related to the device. SEC was not detectable on follow-up imaging in any case in the initial SEC + group and in 1 case in the initial SEC − group (*p* = NS). Preprocedural SEC had no effect on the annual rate of TEs (i.e., combination of nonfatal stroke, TIA or SE) in survivors after hospital discharge: there was a 1.2% event rate in the SEC − group, whereas there was not a single event in the SEC + group (*p* = NS). The combined secondary outcome measure was thus achieved by SEC − 98.8 and SEC + 100.0% of patients, respectively. This was not affected by the statistically significantly different rate of dual antithrombotic therapy at hospital discharge. Estimated 1-year mortality was 12.0% in SEC − and 11.8% in SEC + patients (*p* = NS; Fig. 2).

**Table 3:** Events during follow-up

	SEC −	SEC +	*p* value*
Discharged alive, *n* (%)	501 (99.6)	85 (100.0)	1.00
Information on vital status obtained, *n* (% of all patients discharged alive)	490 (97.8)	85 (100.0)	0.38
Death within 365 days, *n* (% of patients with documented vital status)	56 (11.4)	10 (11.8)	0.86
Events in survivors of follow-up (at 365 days)			
Surviving patients with detailed follow-up information, *n*	404	69	
Major adverse events			
Nonfatal ischemic stroke, *n* (%)	2 (0.5)	0 (0)	1.00
Transient ischemic attack, *n* (%)	2 (0.5)	0 (0)	1.00
Systemic embolism, *n* (%)	1 (0.2)	0 (0)	1.00
Severe bleeding, *n* (%)	3 (0.7)	0 (0)	1.00
Severe groin complication, *n* (%)	1 (0.2)	0 (0)	1.00
Pericardial effusion requiring action, *n* (%)	1 (0.2)	0 (0)	1.00
Device dislodgement requiring action, each *n* (%)			
• Surgical	2 (0.5)	0 (0)	1.00
• Additional intervention	1 (0.2)	0 (0)	1.00
Myocardial infarction, *n* (%)	2 (0.5)	2 (2.9)	0.10
Pulmonary embolism, *n* (%)	6 (1.5)	0 (0)	0.60
Moderate adverse events			
Moderate bleeding, *n* (%)	16 (4.0)	3 (4.3)	0.75
Deep vein thrombosis, *n* (%)	1 (0.2)	0 (0)	1.00
Rehospitalizations			
Total, *n* (%)	140 (39.5)	24 (34.8)	0.50
Device complication-related, *n* (%)	5 (1.4)	0 (0)	1.00
Bleeding-related, *n* (%)	8 (2.3)	1 (1.4)	1.00
AF-related, *n* (%)	9 (2.5)	0 (0)	0.37
Other cardiovascular cause-related, *n* (%)	40 (11.3)	13 (18.8)	0.11

^*^Tested by Pearson’s *X*^2^, Fisher’s exact, or Mann–Whitney-Wilcoxon test; more than one item could occur in the same patient; *AF* atrial fibrillation

Major bleeding after hospital discharge occurred in SEC − 0.7 vs. SEC + 0% of patients (*p* = NS). Pulmonary embolism occurred in SEC − 1.5 vs. SEC + 0% of patients (*p* = NS). At the end of follow-up, the rates of the different antithrombotic regimens no longer differed statistically significantly (Fig. 1B), with only a minority of patients no longer receiving any antithrombotic therapy (SEC − 11.5 vs. SEC + 8.7%, *p* = NS).

## Discussion

We found an overall very low rate of DRT after LAAO, and no association to the presence of SEC at the time of implantation. Likewise, TEs were not more frequent in the group with SEC.

Numerous predictors of DRT were significantly more common in the SEC + group [6]. Most importantly, about twice as many patients were indicated for LAAO because of medication non-compliance. However, in both groups, major bleeding was by far the main reason for contraindication to long-term OAC (in about ¾ of cases this was chosen as reason). In this respect, our study population did not differ from those of other large real-world registries [15, 16]. In addition, patients in the SEC + group showed a trend towards a higher mean CHA_2_DS_2_-VASc score and had significantly larger LAs and LAAs, as well as lower LVEF. Regarding procedure-associated predictors such as a deeper occluder implantation or more paradevice leaks, there was no significant clustering in the SEC + patients. SEC is a long and well-known phenomenon [17], whose causes are not yet fully understood. It is caused by ultrasound reflections on the red blood cells. An increased hematocrit, an increased fibrinogen concentration, increased LA dimensions, and low shear stress could be proven as causative factors [11]. In contrast, a higher-grade mitral regurgitation has a protective effect [14]. SEC is a well-known precursor of thrombus formation in native LA [17, 18]. AF patients with SEC have an increased rate of TEs even in the NOAC era [12], and in addition, strokes are more severe [19]. After LAAO, we could not confirm these adverse effects of SEC in our study group. An overall rate of < 2% for TEs was detectable after hospital discharge, all in the SEC − group, none in the SEC + group. The rate of nonfatal strokes of 0.4% within one year in this LAARGE substudy is consistent with the low rates in other study populations with predominant APAs after LAAO [20]. It needs to be emphasized, that LAARGE registered only nonfatal strokes, fatal events were counted as cases of death [13]. TEs were rare in LAARGE, and the occurrence of only single cases during follow-up was in line with expectations from other registries on LAAO [21, 22]. Although a significant difference between groups was not expected, the nonaccumulation of TEs in the SEC + group is remarkable and should not have been anticipated in advance. Likewise, severe intrahospital complications were rare with only one stroke in the SEC − group. The frequency of MACCE (SEC − 0.6 vs. SEC + 0%) or other major complications (SEC − 3.2 vs. SEC + 4.7%) was within the range of other large clinical registries. For example, the EWOLUTION registry, which also reflects the clinical reality of LAAO, reported a serious adverse event rate within the first 7 days of 4.1% [23]. Within the scope of LAARGE, moderate complications, which do not affect the patient’s long-term outcome but may still cause inconvenience and prolonged hospitalization, are rarely recorded. LAARGE addresses these safety events (predominantly minor bleeding events) in a particularly sensitive way, resulting in an allegedly high rate of about 9%. Device embolisms, TIAs, and infections are represented only in isolated cases and are thus extremely rare, comparable to other large registries [24, 25]. The reason for an accumulation of pericardial effusions not requiring intervention in the SEC + group cannot be named but the increased rate did not derive from a more complex procedure (comparable fluoroscopy time between groups).

On closer view, DRT rates are described from about 0 to just under 10% after LAAO, thus exhibiting a wide range [6, 7]. This implies a multi-factorial causal network. DRTs are associated with a higher rate of TEs after LAAO [26]. Contrary to expectation, it has been repeatedly shown that the postprocedural antithrombotic regimen is not associated with an increased DRT rate [10, 27, 28]. In our study, despite the presence of SEC, AC in addition to APAs was prescribed less frequently compared to patients without SEC at hospital discharge. There might be an indirect explanation for this result, which is not related to SEC: More WATCHMANs™ were implanted in the SEC − group. In the early years of LAARGE, a transient OAC period was often performed in clinical practice after WATCHMAN™ implantation, as this had been envisaged in PROTECT-AF [29]. However, this did not affect TE rates at follow-up. Furthermore, another substudy of LAARGE recently showed no difference in TE rates between patients with AC and APAs [30]. As a result, we found no evidence that more intensive antithrombotic medication with additional AC is required in patients with preexisting SEC. While patient-related DRT risk factors were also present in our study population, the exceptionally low rate of DRTs in LAARGE might be explained by a high procedural quality, reflected by no paradevice leaks > 5 mm after the procedure (and in only 5% of patients on follow-up imaging). In LAARGE, there was a high number of centers where experienced operators performed LAAOs. This may suggest that modifiable risk factors linked to the implantation procedure are crucial for the occurrence of DRT and are preventable by good procedural performance.

In summary, in accordance with previous work [9], preprocedural detection of SEC was associated with the typical risk factors for DRTs and TEs also in LAARGE, but DRTs were not detectable in the SEC + group after LAAO.

## Limitations

This registry is one of the biggest registries following LAAO with a wide range of different commercially available types of devices. Patients participated in LAARGE on a voluntary basis according to ethical standards. This could potentially lead to a selection bias, resulting in variations in age, sex, frailty, multimorbidity, and education level [31]. Although LAARGE reports on individualized rather than standardized therapies, it contains a wealth of data from clinical practice and multicenter experience. Only patients with started LAAO procedure were included in the LAARGE subanalyses. If patients had not been admitted to the LAAO procedure by their treating physician because of preexisting SEC, this would not have been recorded. However, we wanted to investigate the impact of SEC on procedure performance, effectiveness, and safety, so that only the selected patients were relevant for our aim. We recognize that, to date, there is no global, uniform definition of SEC and, therefore, there might have been some variation in the definition of SEC at participating centers. The graduation of SEC was not queried and could not be collected for this substudy. Fatkin and colleagues reported an association of increasing SEC density with decreasing flow velocity in the LAA; however, a positive association with TEs was not found [14]. Nevertheless, nowadays, an association of dense SEC and an increased rate of TEs may be assumed [32]. In this respect, the omission to assess SEC density should be recognized as a relevant limitation. The follow-up period was limited to one year after the procedure, which, however, covers the relevant period for DRT detection [7]. Therefore, late clinical events beyond one year cannot be evaluated. Only clinically overt neurological events were documented, no imaging information regarding cerebral scans during follow-up was gathered in this registry. Nevertheless, we consider the data presented to be of value in their aggregate clinical implication.

## Conclusions

The results show that presence of LA SEC in patients undergoing endovascular LAAO is not associated with increased DRT rate or TE risk at one-year follow-up. The incidence of both entities was very low in this registry despite a high proportion of patients not receiving OAC after the procedure.

### Supplementary Information

Below is the link to the electronic supplementary material.

Supplementary file1 (DOCX 23.1 KB)

## Abbreviations

AF: Atrial fibrillation
APA: Antiplatelet agent
DRT: Device-related thrombosis/thrombus
IHF: Institut für Herzinfarktforschung
LA(AO): Left atrial (appendage occlusion)
LVEF: Left ventricular ejection fraction
MACCE: Major adverse cardiac and cerebrovascular events
(N)(O)AC: (Non-vitamin K) (oral) anticoagulation/anticoagulants
NS: Not significant
SE: Systemic embolism
SEC: Spontaneous echo contrast
TE: Thromboembolic events
TIA: Transient ischemic attack
vs.: Versus
