# Supplementary material for: Is spontaneous echo contrast associated with device-related thrombus or embolic events after left atrial appendage occlusion? - Insights from the multicenter German LAARGE registry
Source: J Interv Card Electrophysiol. 2023 Jun 1;67(1):119–28. doi: 10.1007/s10840-023-01567-z (PMC10770218; doi:10.1007/s10840-023-01567-z)
Supplement: Supplementary file 1 — Supplementary file1 (DOCX 23.1 KB) [file 10840_2023_1567_MOESM1_ESM.docx]

| **Supplementary table 1: Preprocedural imaging data** | | | |
| --- | --- | --- | --- |
|  | **SEC-** | **SEC+** | ***p* value*** |
| **Total cohort, *n* (% of all patients)** | 503 (85.5) | 85 (14.5) |  |
| **LVEF [%], median (IQR)** | 60 (50; 60) | 50 (45; 60) | **< 0.001** |
| **LA diameter [mm], median (IQR)** | 47 (43; 51) | 50 (47; 54) | **< 0.001** |
| **LA surface [cm^2^], median (IQR)** | 26 (19; 31) | 33 (30; 50) | **0.003** |
| **LAA ostial diameter [mm], median (IQR)**   - **0°** - **45°** - **90°** - **135°** | 19 (17, 22)  20 (17; 22)  20 (17; 22)  20 (18; 22) | 22 (19; 23)  21 (19; 23)  21 (18; 23)  21 (19; 24) | **0.001**  **0.011**  0.078  0.051 |
| **LAA morphology, each *n* (%)**   - **cactus** - **cauliflower** - **chicken wing** - **windsock** - **atypical** | 43 (8.8)  76 (15.6)  215 (44.1)  73 (15.0)  80 (16.4) | 8 (9.5)  13 (15.5)  42 (50.0)  16 (19.0)  5 (6.0) | 0.84  0.98  0.32  0.34  **0.013** |
| **Number of lobi, each *n* (%)**   - **1** - **2** - **> 2** | 257 (53.2)  191 (39.5)  35 (7.2) | 48 (57.1)  32 (38.1)  4 (4.8) | 0.43 |
| *tested by Pearson’s *X*^2^ or Mann-Whitney-Wilcoxon test; bold indicates *p* < 0.05; IQR, interquartile range; LA(A), left atrial (appendage); LVEF, left ventricular ejection fraction | | | |

| **Supplementary table 2: Procedural details** | | | |
| --- | --- | --- | --- |
|  | **SEC-** | **SEC+** | ***p* value*** |
| **Total cohort, *n* (% of all patients)** | 503 (85.5) | 85 (14.5) |  |
| **Successful implantation, *n* (%)** | 493 (98.0) | 84 (98.8) | 1.00 |
| **Number of implantation attempts, mean ± SD** | 1.7 ± 1.3 | 1.5 ± 0.9 | 0.55 |
| **Paradevice leak, each *n* (%)**   - **< 3 mm** - **3-5 mm** - **> 5 mm** | 29 (6.0)  21  8  0 | 3 (3.6)  3  0  0 | 0.39 |
| **Type of LAAO device, each *n* (%)**   - **Watchman™** - **Amplatzer™ Cardiac Plug** - **Amplatzer™ Amulet™** - **other device** | 254 (50.6)  120 (23.9)  120 (23.9)  8 (1.6) | 19 (22.4)  31 (36.5)  31 (36.5)  4 (4.7) | **< 0.001**  **0.014**  **0.014**  0.061 |
| **Total duration [min], median (IQR)** | 58 (43; 78) | 65 (54; 86) | **0.008** |
| **Fluoroscopy time [min], median (IQR)** | 10 (7; 15) | 11 (8; 15) | 0.28 |
| **Dose area product [cGy*cm^2^], median (IQR)** | 1858 (712; 4104) | 3125 (1397; 5427) | **0.005** |
| **Sedation type, each *n* (%)**   - **conscious sedation** - **general anesthesia** - **other** - **none** | 423 (84.1)  55 (10.9)  15 (3.0)  11 (2.2) | 67 (79.8)  17 (20.2)  0 (0)  0 (0) | 0.32  **0.016**  0.11  0.17 |
| *tested by Pearson’s *X*^2^, Fisher’s exact, or Mann-Whitney-Wilcoxon test; bold indicates *p* < 0.05; IQR, interquartile range; LAAO, left atrial appendage occlusion; SD, standard deviation | | | |
